# Performance evaluation of inpatient service in Beijing: a horizontal comparison with risk adjustment based on Diagnosis Related Groups

**DOI:** 10.1186/1472-6963-9-72

**Published:** 2009-04-30

**Authors:** Weiyan Jian, Yinmin Huang, Mu Hu, Xiumei Zhang

**Affiliations:** 1 School of Public Health, Health Science Center, Peking University, 38# Xue Yuan Road, Hai Dian District, Beijing, PR China; 2 Health Insurance Office, the Third Medical School, Peking University, 17# Xue Yuan Road, Hai Dian District, Beijing, PR China; 3 Beijing Public Health Information Center, 59# Bei Wei Road, Xuan Wu District, Beijing, PR China

## Abstract

**Background:**

The medical performance evaluation, which provides a basis for rational decision-making, is an important part of medical service research. Current progress with health services reform in China is far from satisfactory, without sufficient regulation. To achieve better progress, an effective tool for evaluating medical performance needs to be established. In view of this, this study attempted to develop such a tool appropriate for the Chinese context.

**Methods:**

Data was collected from the front pages of medical records (FPMR) of all large general public hospitals (21 hospitals) in the third and fourth quarter of 2007. Locally developed Diagnosis Related Groups (DRGs) were introduced as a tool for risk adjustment and performance evaluation indicators were established: Charge Efficiency Index (CEI), Time Efficiency Index (TEI) and inpatient mortality of low-risk group cases (IMLRG), to reflect respectively work efficiency and medical service quality. Using these indicators, the inpatient services' performance was horizontally compared among hospitals. Case-mix Index (CMI) was used to adjust efficiency indices and then produce adjusted CEI (aCEI) and adjusted TEI (aTEI). Poisson distribution analysis was used to test the statistical significance of the IMLRG differences between different hospitals.

**Results:**

Using the aCEI, aTEI and IMLRG scores for the 21 hospitals, Hospital A and C had relatively good overall performance because their medical charges were lower, LOS shorter and IMLRG smaller. The performance of Hospital P and Q was the worst due to their relatively high charge level, long LOS and high IMLRG. Various performance problems also existed in the other hospitals.

**Conclusion:**

It is possible to develop an accurate and easy to run performance evaluation system using Case-Mix as the tool for risk adjustment, choosing indicators close to consumers and managers, and utilizing routine report forms as the basic information source. To keep such a system running effectively, it is necessary to improve the reliability of clinical information and the risk-adjustment ability of Case-Mix.

## Background

One of the characteristics of the health services market is the serious information asymmetry between providers and consumers, including managers [1]. Asymmetric information makes it difficult for consumers and managers to estimate the performance of providers, affecting not only patient choice [2,3] but also government decision making.

To solve this problem, one method is to establish a performance evaluation system to help managers learn more precisely about provider performance through the expert system and evaluation technique, which can assist managers with "rational" decision-making [4-6]. As an important part of health services research, the significance of medical performance evaluation lies not only in the establishment of a better performance supervising system but also through evidence-based health policymaking and the regulation of the health services market.

Currently in China, health reform has entered a crucial stage. However, because problems resulting from information asymmetry of the health services market have not been settled effectively, reform remains unsatisfactory. A major cause is the lack of an effective tool for evaluating medical performance.

For an ideal performance evaluation system, the most important feature is the accuracy of its evaluation results. However, the quality of data from health services performance evaluation is often questionable. As different doctors, departments and hospitals admit different patients, medical inputs and outputs are often considered to be non-comparable among different providers [7,8]. Therefore, "risk adjustment" of evaluated objects before evaluation is the key step to increasing comparability. "Case-Mix" is usually used as the tool for risk adjustment in this process [9-11]. The literature concerning performance evaluation of medical services has dramatically increased in China since 1980. "Key Performance Indicators (KPI)" have been used widely, with the most common indicators being medical cost, LOS and medical quality[12]. Subsequently, many methods including the "Balance Score Card (BSC)" were introduced into the performance evaluation of hospitals [13,14]. However, the reliability of evaluation results has remained questionable without risk adjustment.

Until 2000 there were no use of applying Case-Mix to clinical performance evaluation to eliminate the bias caused by diseases' different attributes [15]. Ning etc. (2001) [16], Xinyan etc. (2002)[17] and Jie etc. (2003)[18] conducted the relative research and practice on health service performance evaluation using different Case-Mix systems. In 2005, the researchers of Peking University managed to develop a set of Diagnosis Related Groups (DRGs) named PKU-DRGs based on the front-page data of medical records (FPMR) from the local hospitals. With this model, we conducted some tentative experiments on hospital performance evaluation in 2006 and 2007 using the FPMR data from some large public hospitals in Beijing. The findings indicated that, after the standardization of PKU-DRGs, evaluation results were more reliable. [19,20]

Until now, research about using Case-Mix as a tool for risk adjustment to evaluate medical performance is still at an early stage in China. The application of results to policy practice is even less developed. In addition, it is also necessary to make the evaluation results direct-viewing and the job convenient. As directly expressed results are easy for users to understand, and thus form the basis for decision-making, the convenience of evaluation can help to maintain continuity and avoid short-term behaviours of evaluated providers. This requires the meaning of evaluation indicators to be clear and concrete [21], and costs to be low. Since the cost of evaluation is often primarily related to data collection, using data from routine report forms can significantly reduce the expense. [22-24].

Based on the current situation of China and our understanding of performance evaluation, and with the aim of making performance evaluation more accurate, direct-viewing and convenient, an appropriate tool for risk adjustment was chosen to establish proper evaluation indicators in this study. Using the data from routine report forms, the performance of public hospitals' inpatient services in Beijing was evaluated, with the aim of accumulating experience to construct a health services evaluation system suitable for China (Beijing).

Health service performance can be evaluated at many levels such as case, disease, case-mix, physician, department and hospital. In this study we chose to analyze at the hospital level.

## Methods

### 1 Data Collection

Evaluation was undertaken at all large public hospitals of general acute care (21 hospitals) in Beijing, excluding chronic hospitals and special hospitals.

At the end of 2006, the Beijing Health Bureau (BHB) carried out a program designed to standardize the information from FPMR. A special norm for writing FPMR was issued by BHB, and doctors and administrators of information systems from all large public hospitals which provide inpatient services in Beijing were trained. From the third quarter of 2007, all evaluated hospitals in this study started to report electronic data of FPMR using a unified standard to Beijing Public Health Information Center (BPHIC) through a special network every quarter.

After standardization, the FPMR involve 108 variables, including general demographic information (age, gender etc.), date of admission and discharge, diagnosis (including principal diagnosis and other relative diagnosis), procedures (including all procedures performed during hospital stay), medical charge and information about hospitalization outcome (discharge, discharge against advice, inter-hospital transfer and death). The data format of variables was regulated by BPHIC. Diagnosis and procedures were coded using the unified International Classification of Disease-Beijing Clinical Modification (ICD-BJCM) also issued by BPHIC.

The data used in this study came from the FPMR of these hospitals in the third and fourth quarter of 2007 collected from BPHIC. The total number of FPMR collected was 254,190. The integrity and accuracy of the FPMR data was examined at BPHIC, and underwent logic error detecting. After error detecting, there were 245,672 FPMR (96.56%) entering the PKU-DRGs program.

### 2 Data Analysis

#### (1) the selection of the tool for risk adjustment

The selection of the tool for risk adjustment has an important effect on the reliability of evaluation results [25]. Since this study only paid attention to general acute care hospitals, we considered the DRGs based on diagnosis and procedures to be the ideal tool for risk adjustment. However, a DRGs developed in China are unavailable, compared to the DRGs of other countries which have been established on the basis of their own clinical practice and data environment. Hospitals in Beijing use unitive ICD-BJCM, with diagnosis codes based on ICD-10 and procedure codes based on ICD-9. Therefore no edition of DRGs from another country could be applied directly for the purposes of this study.

As such, we adopted the PKU-DRGs grouping program developed by Peking University as the tool for risk adjustment. It was designed in accordance with the ICD-BJCM standard issued by BPHIC so that it matched the collected FPMR data. The grouping process of PKU-DRGs is similar to that of other DRGs: first, assign a given case to its Major Disease Category (MDC) according to the primary diagnosis; then tentatively determine the DRG it might belong to on the basis of primary diagnosis and (or) primary procedure; determine sequentially the severity of complication and comorbidity through other diagnoses and procedures; and finally decide the DRG the case should be assigned to, taking into account the patient's individual characteristics (e.g. age, gender, etc.). The PKU-DRGs therefore have a relatively strong ability for risk adjustment because the various factors above are all involved in the grouping process. Moreover, we used this programme in 2006 and 2007 to analyze data from some hospitals in Beijing with good results.

#### (2) establishment of the evaluation indicators

We evaluated the whole performance of hospitals' inpatient services in this study. Work efficiency and service quality of hospitals are key concerns for consumers and managers, and are widely used in performance evaluation [26-28]. This study therefore began from these starting points to establish evaluation indicators with PKU-DRGs risk adjustment.

#### 1) Work efficiency

The dimensions medical expenditure and LOS were used in this study to measure the efficiency of different hospitals. After risk adjustment, hospitals with low medical expenditure and short LOS were considered to be efficient. Using PKU-DRGs as the tool for risk adjustment, the two indices "Charge Efficiency Index (CEI)"and "Time Efficiency Index (TEI)" were established. The specific steps were as follows:

□ calculate charge per case () and average LOS () within each DRG based on full sample size;

□ calculate charge per case () and average LOS () within each DRG in that hospital;

□ calculate the ratio *k *between that hospital and full sample size:

Charge ratio = , LOS ratio = ;

□ Charge Efficiency Index ,

Time Efficiency Index 

in which, *n*_*j *_is the number of cases in *DRG*_*j *_in the hospital.

We then used Case-Mix Index (CMI) [25,29,30] to adjust CEI and TEI.



CMI was calculated using patient's hospitalization charge contained in the FPMR data. The specific calculation was [31]:



in which,

*h *is the hospital for which the index was being calculated;

*W*_*g *_is the weight associated with the *DRG*_*g *_which was calculated by the function as  in this study;

*n*_*gh *_is the number of cases in the *DRG*_*g *_in hospital *h*; and

*n*_*gn *_is the number of cases in the *DRG*_*g *_of the entire sample.

A computed value of *aCEI *or *aTEI *of 1 means that the work efficiency of a hospital is close to average; if less than 1, it indicates that the medical charges of that hospital are lower or LOS shorter than the average; if more than 1, it indicates that the medical charge of that hospital is higher or LOS longer than the average.

#### 2) Service quality

Based on inpatient mortality, "DRGs death risk score" was used here to establish evaluation indicators which to measure the overall medical service quality of a hospital. The death risk of different DRGs can be classified with inpatient mortality of discharge cases. The specific steps were as follows:

□ compute the inpatient mortality of each DRG (*M*_*i*_);

□ take the logarithm of *M*_*i *_(*Ln*(*M*_*i*_));

□ calculate the Mean () and standard deviation (*s*_*i*_) of *Ln*(*M*_*i*_);

□ calculate the death risk score according the definition of death risk grade.

The definition of each "death risk grade" is shown in Table 1. Score "0"means there is not any death case in these DRGs; score "1" means the inpatient mortality is below Mean -standard deviation – "low-risk group (LRG)"; score "2" indicates the inpatient mortality is between Mean and Mean -standard deviation – "medium-to-low risk group (MLRG)"; score "3" shows the inpatient mortality is between Mean and Mean +standard deviation – "medium-to-high risk group (MHRG)"; and score "4" means the inpatient mortality is above Mean +standard deviation – "high risk group (HRG)".

**Table 1:** Death risk grade and its definition

Score	Definition
0	*M*_*i *_= 0
1	
2	
3	
4	

Inpatient mortality relates to both the disease and the clinical course. The cases in the LRG are those with a very low probability of death under general conditions, such as simple appendix resection. Cases in HRG are those with a very high probability of dying, such as malignant tumor.

For high risk cases, mortality is more related to the disease than the clinical course; for low risk cases, the converse applies. In view of this, **medical service quality was evaluated using the inpatient mortality of low-risk group cases (IMLRG)**, with the lower the IMLRG, the higher the service quality.

The statistical significance of difference between each hospital's IMLRG and the 21 hospitals' average was tested respectively. Considering the low mortality in LRG, we used Poisson Distribution Analysis for testing. The probability density function of a Poisson variable is given by [32]:



in which, *μ *is the mean and *X *is the quantity of death cases.

Let  be the average IMLRG of the 21 hospitals, *D*_*h *_and *X*_*h *_are the IMLRG and death cases of the tested hospital respectively.



The probability was calculated using equations as follows [32]:



or



All these statistical analysis were carried out using SAS statistical software. This study involvedneitherhuman bodyor animal experiments or the use of private data.

## Results

### 1 Description of the evaluated hospitals

Basic information of the evaluated hospitals including the number of beds, physicians, nurses and discharge cases is shown in Table 2. They are all large general public hospitals of acute care in Beijing, with 964 beds, 561 licensed physicians and 717 registered nurses each on average.

**Table 2:** Basic information of the evaluated hospitals

Hospital code	Hospital Name	Beds	licensed physicians	registered nurses	discharge cases	DRGs	CMI
A	The Third Hospital, Peking University	1322	745	813	22087	503	1.134
B	The first Hospital, Peking University	1368	678	1216	19891	495	0.937
C	Ren Min Hospital	1221	684	898	18626	493	1.185
D	Shou Gang Hosptial	695	326	345	7858	389	1.001
E	Dian Li Hospital	518	284	322	4800	343	1.101
F	Hua Xin Hospital	501	305	437	6669	391	0.993
G	Ji Shui Tan Hosptial	971	550	707	11812	391	1.451
H	Jian Mei Hosptial	567	322	467	4126	307	1.087
I	Shi Ji Tan Hospital	767	445	606	8184	434	1.059
J	Phoenix Hospital	515	279	366	5337	350	1.014
K	Aerospace Central Hospital	660	309	359	7009	404	0.841
L	An Zhen Hosptial	937	729	775	14983	403	2.087
M	Chao Yang Hospital	1476	886	1038	20025	497	1.083
N	Tian Tan Hosptial	926	636	801	11483	386	1.363
O	Tong Ren Hospital	1286	800	965	19412	450	0.844
P	Friendship Hosptial	892	600	740	12657	326	0.917
Q	Fu Xing Hosptial	557	317	484	5527	330	0.864
R	Xuan Wu Hospital	981	650	701	14858	438	1.195
S	Beijing Hospital	952	565	914	9203	419	1.101
T	Xie He Hospital	1837	978	1197	12670	457	1.078
U	China-Japan Friendship Hospital	1302	698	912	16974	428	0.938

Average		964	561	717	12104	___	___

* Data of beds, licensed physicians and registered nurses came from Beijing Assembly of Health Statistics (2006). "Discharge cases" is the number of cases discharged in the3^rd ^and 4^th ^quarter of 2007. "DRGs" means the number of PKU-DRGs covered by each hospital's discharge cases in the 3^rd ^and 4^th ^quarter of 2007. The values of CMI were calculated using the FPMR data of discharge cases in the 3^rd ^and 4^th ^quarter of 2007.

In the third and fourth quarter of 2007, the number of discharge cases averaged 12104 per hospital. Each one of the 21 evaluated hospitals has a wide rage of case categories. They all have more than 300 DRGs. The hospital with the largest service scope is A (503 DRGs), then M (497 DRGs). 12 hospitals have more than 400 DRGs. Among these 21 evaluated hospitals, L has the highest CMI (CMI = 2.087). The others range from 0.8 to 1.5, 13 of them greater than 1.

### 2 A comparison on work efficiency at hospital level

**Figure 1:**
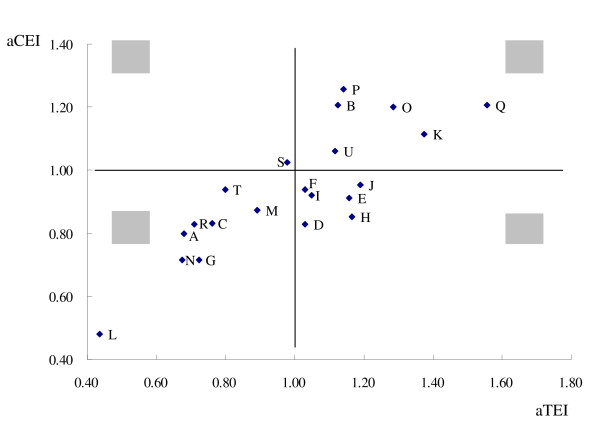
**Distribution of aCEI and aTEI among the 21 hospitals (2007 Q3–Q4)**. This figure is divided into 4 quadrants by two lines (aCEI = 1 and aTEI = 1). The performance of the hospitals in the first quadrant was worse than the other hospitals due to their higher charge and longer LOS. The hospitals in the second quadrant had a longer LOS despite a lower charge compared with the others. The performance of the hospitals in the third quadrant was better than the other hospitals because of their shorter LOS and lower charge. The hospitals in the forth quadrant had a higher charge in spite of a shorter LOS.

The distribution of aCEI and aTEI among the 21 evaluated hospitals is shown in Figure 1. It is divided into 4 quadrants by two lines (aCEI = 1 and aTEI = 1).

The first quadrant contains 6 hospitals (29%) including B, K, O, P, Q and U, the performance of which was worse than the other hospitals due to higher cost and longer LOS.

The second contains hospital S which had higher costs despite shorter LOS compared with the others.

The third includes 8 hospitals (38%) including A, C, G, L, M, N, R and T, the performance of which were better than the other hospitals because of shorter LOS and lower expenditure. Among them L performed best due to its lowest cost and shortest LOS.

The fourth contains 6 hospitals (29%) including D, E, F, H, I and J which had longer LOS and lower cost.

There were 12 hospitals (57%) whose LOS were longer than average and half of them were also more expensive. Low work efficiency seems to be a more widespread problem in Beijing than high cost.

### 3 A comparison on Medical quality at hospital level

**Figure 2:**
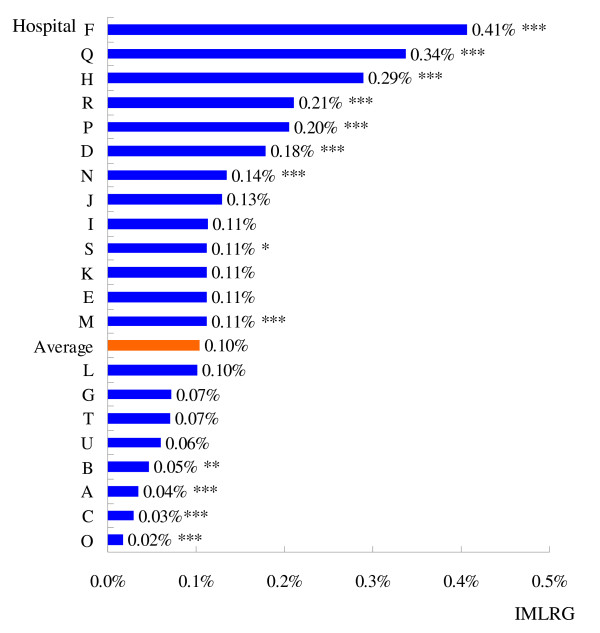
**Inpatient mortality of low risk group for each hospital(2007 Q3–Q4)**. "Average" was obtained through dividing the number of all the cases by the number of the death cases in the LRG. The statistical significance of difference between each hospital's IMLRG and the 21 hospitals' average was tested respectively with Poisson distribution analysis. * Statistically significant at the 10% level; ** statistically significant at the 5% level; *** statistically significant at the 1% level.

In the 245672 cases, 82133 were in LRG with 85 mortality cases. The average IMLRG was 0.10%. These mortality cases were distributed among the 21 hospitals. The IMLRG for each hospital is shown in Figure 2. Among them Hospital F had the highest IMLRG (0.41%), and Hospital O the lowest IMLRG (0.02%). The former was 20 times higher than the latter.

Using Poisson distribution analysis, at the statistically significant 10% level, 13 hospitals (A, B, C, D, F, H, M, N, O, P, Q, R and S) showed a significant difference to the mean. The IMLRG of hospitals A, B, C and O were significantly lower than the average (0.1%) while of the IMLRG of hospitals D, F, H, M, N, P, Q, R and S were significantly higher. This evaluation showed that 43% of these 21 hospitals had relatively poor medical service quality, 38% were moderate and only 19% performed relatively well.

Through the horizontal comparison among the local hospitals of the same type, the performance distribution of each hospital in Beijing can be clearly seen. Using the locally developed DRGs system as the tool for risk adjustment, we found that Hospital A and C had a relatively good overall performance because their medical charges were lower, LOS shorter and IMLRG smaller. The performance of Hospital P and Q was the worst due to their relatively high charge level, long LOS and high IMLRG. Various performance problems also existed in the other hospitals.

## Discussion

### 1 Characteristics of this performance evaluation

The aims of performance evaluation inform the selection of indicators and the evaluation method. We tried in this study to develop a medical performance evaluation system which can be understood and used by non-professional managers. So a series of "simple" but definite evaluation indicators were established.

To address the "comparability" problem in utilizing these "simple" indicators above directly to compare horizontally among different hospitals, PKU-DRGs – the locally developed Case-Mix system of Beijing – was used as a tool for risk adjustment, to match the data environment of Beijing.

In this study, the analysis of each performance indicator was completed based on FPMR electronic data routinely collected in public hospitals and reported electronically every quarter with unified variable type and data format. Evaluating performance with these data avoids the cost and complexity of new data collection. More importantly, as these data are accumulated continuously, continual evaluation and historical analysis to promote the continuous improvement of hospital performance is possible.

Evaluation results were generated using "relative value" in this study (e.g. not using the absolute value of medical charges and LOS, but instead calculating CEI and TEI). The aim in doing so was to promote the continuous improvement of medical service performance. Public dissemination of these results would motivate hospitals to improve their performance, especially for those ranking low. When trying to improve performance, hospitals wanting to improve ranking would need to improve faster than the average. Even relatively high performing hospitals would also have to make efforts to improve their performance.

Performance evaluation of inpatient services was designed for accuracy, direct view and convenience. PKU-DRGs were introduced as the tool for risk adjustment to resolve the problem of accuracy; using clear and definite indicators. Ranking the hospitals with relative value made the evaluation results more direct-viewing. For the sake of convenience, the FPMR as a routine report form was used here as the basic information source to reduce cost and improve the continuity of performance evaluation.

### 2 Factors that may affect the reliability of the results

Coding accuracy is one factor that may affect results reliability. A wrong code may lead to the inappropriate assignment of DRG. If in a hospital, such mistakes do not happen accidentally but systematically, a considerable number of cases will be assigned to inappropriate DRGs whose weights are not fit for these cases. This would directly affect the computation of CMI, aCEI and aTEI for this hospital.

Data integrity is another concern. All the node variables related to DRG grouping could influence the results. BPHIC has a special quality control measure for the integrity of the FPMR data. In this study, data such as age, gender, etc. are verified. Yet, for secondary diagnosis and procedure, the integrity will be much more difficult to control. If the secondary diagnoses are absent from the data provided by a hospital, the cases which should be assigned to DRGs with complications and comorbidity may be improperly assigned to those without complication and comorbidity, which could potentially lead to underestimates of the hospital's CMI.

Thridly, the hospitals selected in this study are all large general hospitals which admit all kinds of cases (the cases admitted by Hospital H, which has the smallest admission range, covered 307 DRGs). However, some of these hospitals still have their own clinical specialty. Such specialized cases may account for a large proportion of their admissions. Such hospitals therefore may not be validly compared in performance with other hospitals.

Hospital L in this study had a very high CMI (2.087). Its performance turned out to be highly efficient (aCEI = 0.479, aTEI = 0.434) after the adjustment with CMI. One possible reason for this might be the better data integrity of Hospital L. But its high proportion of specialized cases could be the most important factor.

In addition, since the number of mortality cases is very small in LRG, the sensitivity of index IMLRG needs to be tested. The low incidence events conform to a Poisson distribution, so Poisson distribution analysis was used in this study to test the significance of difference between each hospital's IMLRG and the total average. It should be noted that the sample size can influence the result of significance test. In this study, the IMLRG values of Hospital E, I, K, M and S were fairly close to one another (all at about 0.11%). But in the significance test, Hospital M and S were statistically significant while the other three were not. It was found that the number of LRG cases in Hospital M and S was much more than the other three. If a larger volume of data could be accumulated (e.g. 1 or 2 years), the sensitivity of the Poisson distribution test would be even higher.

### 3 The use of evaluation results

Whether or not performance evaluation improves performance firstly depends on whether and how evaluation results are applied [33]. Inappropriate use is unlikely to improve performance, and instead, may force hospitals to resort to deception in data reporting and even refuse high-risk patients.

The objective of this evaluation was not to reward or punish the evaluated providers, but to help the government and funders understand the performance of different hospitals and allow these hospitals to improve their performance. To achieve this goal, firstly, results need to be known by the hospitals with their relative position known and performance goals established; and secondly, evaluation needs to be repeated and sustained with the same methodology. Horizontal and historical analysis should be conducted using the results so that the improvement of hospital performance can be evaluated.

This evaluation used hospitals as evaluation units. The results can assist macro regulators to comprehend the overall performance of the hospitals in Beijing and also help hospital managers learn their performance ranking in the city. However, for a given hospital, the specific roots of performance problems still may not be easily identified. The results can indicate where problems may lie, but they do not give the solution. Therefore, to get more applicable conclusions, it is necessary to analyze the specific cases of that hospital further, and even go to the hospital to do field research. This job can be carried out aiming at specific problems of specific hospitals after the macro results of performance evaluation are available. Only in this way can the performance evaluation play a real role in promoting hospitals to improve their performance.

### 4 The limitation of this study and issues that need further research

There are several potential limitations of this study. Firstly, the PKU-DRGs used in this study as a tool for risk adjustment were only developed recently. So their suitability for risk adjustment still needs to be tested and improved in the future.

Secondly, although the FPMR data was standardized by BHB and BPHIC and the physicians and the information administrators trained in its use, it is still very difficult to judge whether the hospitals reported the data with sufficient quality. It is even more difficult to judge whether they modified the data in any way, for example through upcoding. However, as the hospitals in Beijing are still using fee-for-service, upcoding cannot help them to increase their income directly. We thus believe that the problem of upcoding is not significant.

Another potentially important modification of data is the withholding of information which could reflect adversely on the hospitals, such as consistency of diagnosis, bedsores and iatrogenic infection. Evaluate medical quality merely using IMLRG may thus be insufficient as these factors are all important quality indicators. Nevertheless, because the FPMR data were reported by the hospitals themselves, it was difficult to assess the error of these indices. Therefore they were not used in this study.

Thirdly, the charge-based CMI was used to evaluate the severity of illness and adjust efficiency indices in this study. The adjustment ability of CMI was weakened to a certain extent, because charge can not fully reflect the severity of illness, especially, when the service prices of public hospitals are controlled by the government in China.

In the future, it is necessary to make efforts to improve and update PKU-DRGs, while also maintaining the standard and improving the reliability of FPMR data through supervision and checks. More evaluation indicators for medical service quality should be added on the basis of accurate data. Considering the limitation of charge-based CMI, expert rating could be used to assign weight to DRGs, to correct the deviation caused by charge-based weighting.

## Conclusion

Using Case-Mix as the tool for risk adjustment, choosing the indicators that are close to consumers and managers, and utilizing the data from routine report forms as the basic information, it is possible to develop an accurate and direct-viewing performance evaluation system which is easy to run continuously. The proper utilization of the results from this system can help third party and regulators understand the performance of different hospitals, which could contribute to reducing problems resulting from information asymmetry in the medical market.

The ability of Case-mix to undertake risk adjustment and the reliability of routine data are key to ensuring the reliability of evaluation results. Therefore, it is necessary to increase investment in information systems in hospitals, while also improving the tool of Case-Mix and establishing a mechanism to assess the reliability of the data from hospital report forms. Only by establishing an effective performance evaluation system of medical services and continually monitoring hospital performance, can the medical market enter a virtuous circle.

## Abbreviations

(DRGs): Diagnosis Related Groups; (PKU-DRGs): Diagnosis Related Groups developed by Peking University; (FPMR): The front pages of medical records; (BPHIC): Beijing Public Health Information Center; (ICD-BJCM): International Classification of Disease-Beijing Clinical Modification; (CMI): Case-mix Index; (CEI): Cost Efficiency Index; (TEI): Time Efficiency Index; (LRG): Low-risk group; (MLRG): Medium-to-low risk group; (MHRG): Medium-to-high risk group; (HRG): High risk group; (IMLRG): Inpatient mortality of low-risk groups cases.

## Competing interests

The authors declare that they have no competing interests.

## Authors' contributions

WJ prepared the data, performed the statistical analysis and drafted the manuscript. YH participated in the statistical analysis. MH participated in the design of the study.XZ conceived of the study, and participated in its design and coordination. All authors read and approved the final manuscript.

## Pre-publication history

The pre-publication history for this paper can be accessed here:


